# Therapeutic Effects of Specialized Pro-Resolving Lipids Mediators on Cardiac Fibrosis via NRF2 Activation

**DOI:** 10.3390/antiox9121259

**Published:** 2020-12-10

**Authors:** Gyeoung Jin Kang, Eun Ji Kim, Chang Hoon Lee

**Affiliations:** 1 Lillehei Heart Institute, University of Minnesota, Minneapolis, MN 55455, USA; kang0268@umn.edu (G.J.K.); kim00662@umn.edu (E.J.K.); 2 College of Pharmacy, Dongguk University, Seoul 04620, Korea

**Keywords:** cardiac fibrosis, NRF2, lipoxins, resolvins, maresins, neuroprotectins

## Abstract

Heart disease is the number one mortality disease in the world. In particular, cardiac fibrosis is considered as a major factor causing myocardial infarction and heart failure. In particular, oxidative stress is a major cause of heart fibrosis. In order to control such oxidative stress, the importance of nuclear factor erythropoietin 2 related factor 2 (NRF2) has recently been highlighted. In this review, we will discuss the activation of NRF2 by docosahexanoic acid (DHA), eicosapentaenoic acid (EPA), and the specialized pro-resolving lipid mediators (SPMs) derived from polyunsaturated lipids, including DHA and EPA. Additionally, we will discuss their effects on cardiac fibrosis via NRF2 activation.

## 1. Introduction

Cardiovascular disease is the leading cause of death worldwide [1]. Cardiac fibrosis is a major factor leading to the progression of myocardial infarction and heart failure [2]. Cardiac fibrosis is characterized by the net accumulation of extracellular matrix proteins in the cardiac stroma and ultimately impairs cardiac function [3]. Therefore, interest in substances with cardioprotective activity continues. It has been emphasized that antioxidant activity by nuclear factor erythropoietin 2 related factor 2 (NRF2) is essential for cardiac protection by unsaturated essential fatty acids such as DHA and EPA. Recently, there are increasing reports that NRF2 is regulated by specialized pro-resolving lipids (SPMs) originated from docosahexanoic acid (DHA) and eicosapentaenoic acid (EPA). However, there are not many reports on the effect of NRF2 regulation on cardiac fibrosis by SPMs. Thus, in this review, we would like to summarize the relationship between SPMs and NRF2 in cardiac fibrosis so far.

## 2. Cardiac Fibrosis and Its Mediators

### 2.1. Cardiac Fibrosis

Cardiac fibrosis is a natural compensatory process that occurs before the symptoms of heart failure. This is indicated by changes in the structure of the ventricles with increased volume and altered chamber configuration. This condition exhibits several characteristic histological features, such as cardiomyocyte hypertrophy and apoptosis, myofibroblast proliferation, and extracellular matrix (ECM) alterations.

**Figure 1:**
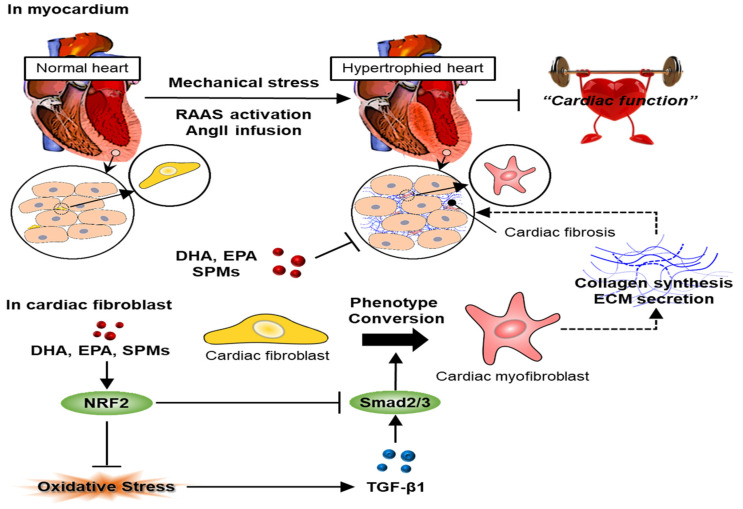
Schematic diagrams depicting the mechanisms of SPMs in alleviating cardiac fibrosis in Ang-II-infused mice. Irisin activated and accelerated the translocation of Nrf2 to the nucleus, thereby reducing oxidative stress and countering the ROS/TGF-β1/Smad2/3 pro-fibrotic pathway (modified from Chen et al. [6]).

Cardiac fibrosis occurs when fibroblasts are activated into myofibroblasts, and the amount of ECM protein increases, altering scar tissue formation and the remodeling of the ECM [4] (Figure 1). Collagen accumulated through these processes affects both systolic and diastolic function and continuously reduces heart function [3]. Cardiac fibrosis is part of the normal ageing process. However, this process is accelerated by many cardiovascular diseases, including diabetes, high blood pressure, and myocardial infarction (MI), as well as ischemic, dilated, and hypertrophic cardiomyopathy [5].

Cardiac fibrosis can be divided into three types. Regional fibrosis (also known as reparative fibrosis) occurs when the affected area is replaced by scar tissue after cardiomyocyte death. Myocardial infarction (MI) occurs due to the prolonged death of cardiomyocytes following a coronary artery occlusion. MI causes a wound healing process characterized by excessive inflammatory reactions and scarring [7]. Forming an appropriate replacement scar at the site of infarction and cardiomyocytes necrosis is an essential response to maintaining the structure of the heart and preventing myocardial rupture. Moreover, diffuse fibrosis (also known as reactive fibrosis) occurs when the affected area is replaced by scar tissue after cardiomyocytes death. This type of fibrosis can be stimulated by pro-fibrotic mediators or prolonged stress, even in the absence of specific cardiomyocyte death. Conditions that apply constant pressure to the heart, such as aortic stenosis or systemic hypertension, have been shown to increase the wall stress in the left ventricle and promote reactive fibrosis in the chamber.

Pathophysiological fluctuations that can trigger the inflammatory response of the heart, such as obesity, diabetes, metabolic syndrome, heart infections, and drugs, cause systemic or local fibrosis [8]. Additionally, invasive interstitial fibrosis leads to the gradual deposition of insoluble proteins (amyloidosis) or lipid hyperglycosylation (Anderson-Fabri’s disease) in the heart stroma [9]. In a normal heart, a well-defined fibrous network helps to convert the function of individual cardiomyocytes into an effective organ pump. However, the ECM, which is overproduced and deposited in pathological conditions, can interfere with the normal electrical conduction pathway between individual cardiomyocytes, which can adversely affect the heart’s contractile performance. Additionally, excess fibrous tissue in the heart affects the transfer of the power of individual cardiomyocytes to a powerful, well-tuned pump function [10]. The accumulation of fibrous tissue around the coronary vessels in the heart’s small myocardium (i.e., perivascular fibrosis) can lead to localized micro ischemic sites, further impairing heart function [11]. Additionally, excessive collagen production in the ECM can affect diastolic function by impairing the elastic rebound of the myocardium as the myocardial cells relax. The accumulation of global or local ECM in the heart can lead to arrhythmia through reentry and other mechanisms [12].

### 2.2. Mediators of Cardiac Fibrosis

Increases in various circulatory hormones, cytokines and proteins due to stress or injury lead to fibroblast activation and differentiation and contribute to cardiac fibrosis [2]. Several studies have confirmed that various substances are involved in this process, suggesting that the renin-angiotensin system (RAS), transforming growth factor (TGF)-beta, endothelin (ET), and inflammation are key. Among them, inflammation will be the focus of the discussion [13].

To briefly explain factors other than inflammation, extensive pieces of evidence, based on various studies, link neurohormonal pathways to the pathogenesis of cardiac fibrosis. The activation of RAS is often found in the fibrotic heart and is associated with the regulation of fibroblast production and activity. Macrophages and fibroblasts that penetrate the damaged heart produce renin and angiotensin-converting enzymes (ACE) and induce the production of angiotensin II (AngII) [14]. Locally released AngII stimulates cardiac fibroblast proliferation and enhances collagen synthesis activity through angiotensin II subtype 1 receptor (AT1R) receptor-dependent interactions [15]. On the other hand, AT2R acts as a negative regulator of the pro-fibrotic reaction. It inhibits AT1R-mediated pathophysiological effects and exerts anti-fibrotic, anti-proliferative, and anti-inflammatory effects [16].

Aldosterone is also known as a mediator of fibrosis [17]. It is known to mediate fibrosis through the stimulation of various cell types. The activation of cytokine and chemokine expression in vascular cells, induction of fibrotic phenotype in macrophages, activation of fibrosis signals in cardiomyocytes, induction of fibroblast proliferation, and increased collagen synthesis have been proposed as mechanisms mediating fibrosis [18].

TGF-β1 is the most characteristic fibrogenic growth factor to date, and in mammals, TGF-β1 exists in three isotypes (TGF-β1, 2, and 3) and is encoded by different genes. Of these, TGF-β1 is the predominant isoform in the cardiovascular system, and so far, most of the information related to cardiac fibrosis is limited to TGF-β1 [19]. Experimental models and previous studies of the human fibrotic heart have shown that TGF-β1 expression increases and continues to be activated during cardiac fibrosis [20]. The activation of TGF-β1 may include heart damage, the production of reactive oxygen species, AngII, hyperglycemia, pH changes and specific proteases, such as plasmin, matrix metalloproteinase-2 (MMP-2), and MMP-9 [21,22]. Activated TGF-β1 has a potent and diverse effect on many cell types associated with cardiac fibrosis, such as macrophages, lymphocytes and cardiomyocytes. TGF-β1 may increase ECM production by stimulating fibroblasts, inducing transformation into myofibroblasts [23]. TGF-β1 also reduces ECM degradation by regulating levels of protease inhibitors, such as Plasminogen Activator Inhibitor (PAI)-1 and Tissue Inhibitors of Metalloproteinases (TIMP) [24]. Besides, TGF-β1 was involved in the synthesis and secretion of other pro-fibrotic factors, such as connective tissue growth factor (CTGF; also known as CCN2) [4].

ET is a protein secreted by heart endothelial cells and is known to play an essential role in the pathogenesis of chronic heart failure [4]. ET has three isotypes (ET-1, 2, 3), of which ET-1 is generally produced in endothelial cells and can be expressed in various cells, such as cardiomyocytes and fibroblasts [25]. ET-1 is known to increase ECM production in fibroblasts, as well as promote the differentiation of fibroblasts into myofibroblasts [26]. Various previous studies have shown that ET-1 is synergistic with other pro-fibrotic agonists [4]. For example, AngII increased ET-1 expression through ERK and reactive oxygen species, and TGF-β1 induced ET-1 through the c-Jun N-terminal kinase (JNK) [27].

Inflammatory cytokines or chemokines can also directly or indirectly affect heart fibrosis. During heart injury, inflammation-related factors are released by cardiomyocytes or fibroblasts [28]. In particular, circulating levels of tumor necrosis factor-α (TNF-α), interleukin-6 (IL-6), IL-2, and IL-1β are known to correlate with disease severity in heart failure patients [29]. Additionally, many T helper 2 (Th2) cytokines, such as IL-4 and IL-13, were first recognized as mediators of pro-fibrotic mediators. IL-4 is known to increase collagen and matrix protein synthesis in fibroblasts, and IL-13 is also known to play a role in fibrosis by directly stimulating fibroblasts [30]. Additionally, the direct and indirect fibrosis properties of IL-17, a Th17 cytokine, have been reported [31].

### 2.3. Cellular Interaction in Cardiac Fibrosis

#### 2.3.1. Cardiac Fibroblasts and Myofibroblasts

Most extracellular materials are secreted by cardiac fibroblasts and make up about 90% of the cells that make up the adult heart. Collagen, one of the crucial proteins secreted into the cardiac extracellular matrix, connects to form fibrous tissue, surrounds the cardiomyocytes, acts as structural support, and has a profound effect on the relaxation of the heart. Cardiac fibroblasts differentiate into myofibroblasts during heart disease in response to certain stress factors, and the amount and composition of extracellular secretions are greatly affected by the proliferation and differentiation of cardiac fibroblasts [32]. Vascular endothelial cells also can differentiate into “active fibroblasts” (i.e., myofibroblasts). Myofibroblasts overproduce and secrete extracellular matrix components, such as collagen, and the expression of intracellular alpha-smooth muscle actin (α-SMA) exhibits the typical features of contractile cells [33]. Additionally, myofibroblasts promote a vicious cycle of fibrosis through their sustained proliferation, self-secretion of TGF-β1 and AngII, expression of AngII type I receptors, and activation of anti-apoptotic genes [34].

#### 2.3.2. Inflammatory Cells

The heart of healthy adult mice contains various types of immune cells such as mononuclear phagocytes, neutrophils, B cells, and T cells. They are contained at a frequency of about 12 times higher than that of skeletal muscle [35]. Cardiac macrophages, similar to macrophages in other tissues, act as guardians to monitor the cause of damage and infection [13]. Cardiac macrophages are energy-intensive and can act as members of a network of steady-state cells needed to maintain the main functions of the heart, which are mechanically dynamic. Moreover, cardiac macrophages can interact electrically with cardiomyocytes by forming gap junctions containing connexin 43 (CX43; GJA1) with cardiomyocytes in addition to typical macrophage activity [36]. In a damaged heart, various macrophage populations, including cardiac resident macrophages and bone marrow progenitor-derived macrophages, may be involved in the initiation, maintenance, and resolution of fibrotic reactions [37]. Similar to the modification of the macrophage phenotype in the normal inflammatory response, during the onset of cardiac inflammation, macrophages acquire a “classic activated M1 phenotype” and release the pro-inflammatory cytokines such as IL-1β, IL-6, and TNF-α. In later stages, macrophages acquire a restorative phenotype through phenotypic transformation and produce anti-inflammatory cytokines, chemokines, AngII, and growth factors [38].

On the other hand, “alternatively activated M2” macrophages regulate the decomposition of extracellular matrix components through MMP release, activate cardiac fibroblasts by secreting TGF-β1, and induce differentiation into myofibroblasts that secrete collagen [39]. Many macrophages accumulate in areas where heart damage has occurred, which can directly contribute to scar collagen production. The overexpression of the C-C Motif Chemokine Ligand 2 (CCL2), which plays an essential role in macrophage recruitment, has been demonstrated to be associated with increased macrophage invasion, extended remodeling, and fibrosis of the heart muscle [40].

Mechanisms that influence macrophage activation include efferocytosis, damage-related molecular pattern (DAMP) production, hypoxia, and ECM remodeling [41]. Cardiomyocyte death under stress conditions, such as severe infarction or prolonged ischemic injury, promotes a phenotype of macrophages dedicated to removing dead cell debris. After swallowing dead cell phagocytes, macrophages secrete TGF-β1 and IL-10 and reduce IL-12 release, resulting in pro-regenerative/fibrotic activity [42]. However, despite the removal of dead cell debris by macrophages, persistent dead cell debris increases the secretion of DAMP and causes a persistent inflammatory response. DAMP activates macrophages through the Toll-like receptor 4 (TLR4) signaling cascade, known as the major inflammatory signaling pathway in macrophages, TLR4/TLR6-IRAK4/1 signaling increases cardiac oxidative stress, and the NLRP3 inflammasome activates the production of IL-1β [43].

Mast cells (MCs) are best known as the primary cells that mediate the anaphylactic reaction in allergies and are characterized by cytoplasmic secretory granules equipped with effector molecules that can be released immediately upon activation. These include molecules, such as various types of chemokines and cytokines, proteases, heparin, histamine, growth factors, and fatty acid metabolites [43]. Pathogen-associated molecular patterns (PAMPs) and DAMPs, which can result from damaged or infarcted cardiomyocytes under stress conditions, can mediate pattern recognition receptors (PRRs) expressed in MCs to induce MCs activation. MC is present in the heart or is recruited to the heart under stress. Although the number of MCs accumulating in the heart is small, it secretes renin, chymase, and tryptase by various pro-fibrotic stimuli to stimulate cardiac fibroblasts and promote fibrosis [44].

The heart muscle is made up of a variety of cell types, and cardiomyocytes make up about 25% to 35% of the cells that make up the adult heart muscle, but 90% of the adult heart’s mass [45]. Cardiomyocytes begin to differentiate and multiply from cardiac progenitor cells during the early stages of heart development, and fetal heart growth is attributed to cardiomyocyte proliferation. However, in the adult heart, cardiomyocytes exit the cell cycle and are known to have minimal cell turnover [45]. Cardiomyocyte death under pathological conditions triggers an inflammatory reaction and promotes fibroblast activation and the production of fibrous tissue to replace dead cardiomyocytes [45]. During this process, large numbers of DAMP signaling factors such as high mobility group proteins B1 (HMGB1), ATP, S100A8-S100A9, and DNA are released from dead cardiomyocytes. Besides, peripheral cardiomyocytes can contribute to interstitial fibrosis by activating interstitial fibroblasts under stress conditions. However, the specific molecular mechanism is not yet clear. Cardiomyocytes secrete IL-6 to induce fibroblast proliferation, and CTGF and VEGF are secreted by protein kinase A stimulation, and collagen production and fibroblast proliferation are induced [46]. Cardiomyocytes also secreted micro-RNA-like molecules in co-culture with cardiac fibroblasts and stimulated collagen production through the activation of the TGF-β1 pathway [47].

## 3. Role of NRF2 in Cardiac Fibrosis

Oxidative stress plays an essential role in the etiology of heart disease. The NRF2/Keap1/ARE pathway, the center of our body’s antioxidant system, is of increasing importance in the treatment of heart diseases, especially heart fibrosis [48,49,50]. We will first describe what NRF2 is and discuss its role in cardiac fibrosis.

### 3.1. NRF2

NRF2 is a transcription factor encoded by the NFE2L2 gene in humans [51]. NRF2 is a basic leucine zipper (bZIP) protein that regulates the expression of an antioxidant protein that protects against oxidative damage from injury and inflammation [52]. NRF2 is at the heart of a complex regulatory network and plays many essential roles in the regulation of metabolism, inflammation, autophagy, protein retention, mitochondrial physiology, and immune responses [53]. Several drugs that stimulate the NFE2L2 pathway are being studied for the treatment of diseases caused by oxidative stress [54].

**Figure 2:**
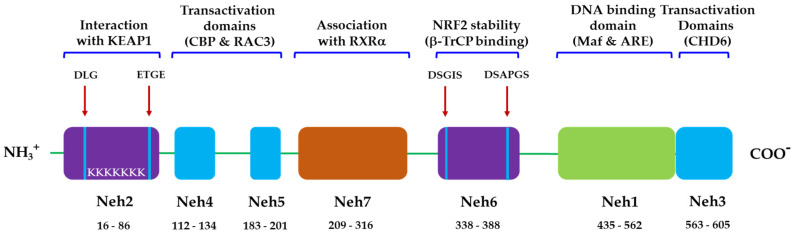
Domain structure of NRF2. Functional Neh domains (amino acid positions): Neh1 (16–86) is the binding site for small Maf proteins and ARE. Neh2 (435–562), the binding site for Keap1 with low-affinity DLG and the high affinity ETGE motifs. Neh3 (653–605), Neh4 (112–134), and Neh5 (183–201) are transactivation domains for NRF2 (CHD6, CBP, and RAC3). Neh6 (338–388) is a serine-rich domain that negatively regulates NRF2 stability by β-TrCP interaction with DSGIS and DSAPGS motifs. Neh7 (209–316) interacts with RXRα, a nuclear receptor responsible for the suppression of the NRF2/ARE signaling pathway.

NRF2 is a basic leucine zipper (bZip) transcription factor with a Cap “n” Collar (CNC) structure [51]. NRF2 has six highly conserved domains called the NRF2-ECH homology (Neh) domains (Figure 2). The Neh1 domain is a CNC-bZIP domain that allows NRF2 to heterodimerize with small molecular weight Maf proteins (MAFF, MAFG, MAFK) [55]. The Neh2 domain allows NRF2 to bind to the cytoplasmic inhibitor Keap1 [56]. The Neh3 domain can play a role in NRF2 protein stability and can act as a trans-activating domain that interacts with components of the transcription device [57]. The Neh4 and Neh5 domains also act as transactivation domains but bind to other proteins, called cAMP-reactive element binding proteins (CREB) that have unique histone acetyltransferase activity [56]. The Neh6 domain may contain degrons, which are involved in the process of NRF2 degradation, which is not sensitive to redox. NRF2 degradation also occurs in stressed cells, which typically prolongs the half-life of the NRF2 protein compared to unstressed conditions by inhibiting other pathways of degradation [58]. When encountering stress conditions, such as oxidative stress, NRF2 is activated by our body’s defense mechanism and enters the nucleus, increasing the transcription of antioxidant-related genes, heme oxygenase-1 (HO-1), NAD(P)H quinone oxidoreductase 1 (NQO-1), superoxide dismutase (SOD), and thioredoxin (TXN).

### 3.2. Role of NRF2 in Cardiac Fibrosis

Some examples of experimental evidence that demonstrates the potential involvement of NRF2 in cardiac fibrosis will be discussed below. Additionally, we will discuss the role of pharmacological activation of NRF2 in reversing many pathological processes caused by diabetic cardiomyopathy.

The treatment of PM 2.5, an air pollutant, in Nrf2-deficient mice exacerbates cardiomyopathy by enhancing oxidative stress, fibrosis and inflammation through RIPK3-regulated mitochondrial disorders [59]. Knockout of Nrf2 (Nrf2 (-/-)) did not cause apparent structural and functional abnormalities in the unstressed heart. However, after transverse aortic constriction (TAC), Nrf2 (-/-) mice developed pathological cardiac hypertrophy, severe myocardial fibrosis and apoptosis, overt heart failure, and increased mortality, which was associated with increased myocardial levels of 4-hydroxy-2-nonenal and 8-hydroxydeoxyguanosine, coupled with the complete inhibition of several antioxidant genes in the myocardial tissue [60].

Three microRNAs (miRs), including miR-27a, miR-28-3p, and miR-34a, were highly expressed in the left ventricle of the heart where myocardial infarction occurred compared to other organs, and cultured cardiomyocytes and fibroblasts responded to TNF-α stimulation, leading to the expression of these three miRs. These miRs are preferentially integrated into exosomes, and miR-rich exosomes appear to contribute to intercellular communication and impaired NRF2 regulation [61]. miR-155 is upregulated in cardiomyocytes in cardiac fibrosis. miR-155 inhibitors significantly restored NRF2 and HO-1 expression levels in hyper glucose-induced cardiac fibrosis, reduced oxidative stress levels, mitochondrial damage, and the number of cells undergoing apoptosis. Additionally, miR-155 inhibitors improved fibrosis by significantly reversing the expression levels of collagen I and α-SMA [62]. Irisin, a hormone released by muscle cells, suppresses the ROS/TGF-β1/Smad2/3 signaling axis through NRF2 to relieve angiotensin II-induced cardiac fibrosis [6] (Figure 1).

Research results on the improvement of cardiac fibrosis by the activation of NRF2 by natural substances have also been reported one after another. For example, puerarin shows the therapeutic effects against AngII-induced cardiac fibrosis. Puerarin prevents cardiac fibrosis via the activation of NRF2 [63]. UGTA1, a metabolic enzyme of puerarin, is increased by NRF2, and puerarin-7-O-glucuronide, produced by UGT1A1, also exhibits similar therapeutic effects to puerarin. Galanthamine activates the AMPK/NRF2 pathway in mice, improving cardiac dysfunction due to myocardial ischemia-reperfusion, endoplasmic reticulum stress-related cell death, and myocardial fibrosis [64]. Isorhynchophylline, an alkaloid derived from *Uncaria rhynchophylla*, enhanced NRF2 in phenylephrine-induced cardiac hypertrophy [65]. Mangiferin degrades Keap1 to enhance stability, promoting NRF2 protein accumulation, leading to NRF2 activation.

By activating NRF2, mangiferin promoted the synthesis of glutathione (GSH) in cardiac fibroblasts, thereby reducing the supply of glutamate to fibroblasts to inhibit activation. Through this action, mangiferin improved cardiac fibrosis induced by transverse aortic constriction [66]. Luteolin prevented cardiac fibrosis, hypertrophy, and dysfunction in streptozotocin-induced diabetic mice through activation of NRF2 and inhibition of nuclear factor-kappa B (NF-κB) [67].

## 4. Specialized Pro-Resolving Lipid Mediators and Their Receptors

The possible involvement of NRF2 in the cardioprotective action of DHA and EPA has been reported. Moreover, SPMs are created from DHA and EPA to induce inflammation resolution. Various physiological and pharmacological actions by DHA and EPA are also likely to be mediated by NRF2. In this section, we will explore these possibilities. Before that, we will briefly introduce what SPMs are and what types of receptors mediate their actions. Furthermore, we would like to present the action they have on NRF2.

### 4.1. Types of Specialized Pro-Resolving Lipids

**Figure 3:**
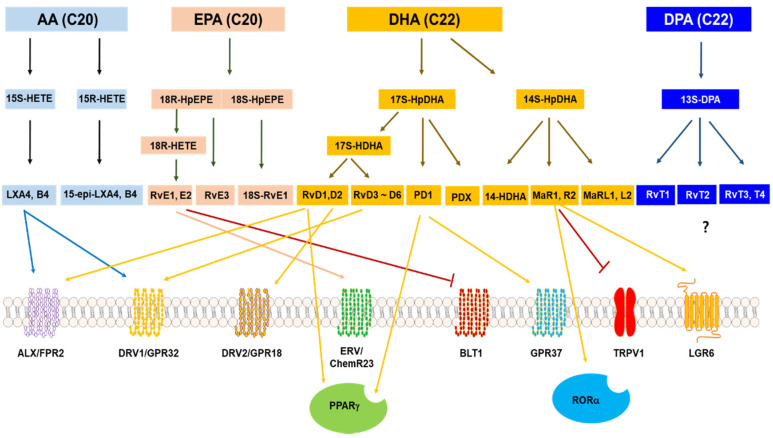
SPMS and their receptors. Arrow heads of lines represent activation of receptors, and bar heads of lines indicate the inhibition of receptors? Means that no receptors are reported for each SPM (modified from Pirault et al. [68]).

SPMs include lipoxins (LXs) made from AA, resolvins (Rvs) made from DHA and EPA, maresins (MaRs), and neuroprotectins (NPD) derived from DHA (Figure 3).

#### 4.1.1. Lipoxins (LXs)

The biosynthesis of LXs differs from other SPMs originated from DHA and EPA in that LXs are produced from arachidonic acid (AA). It is a unique feature that allows different intercellular metabolic pathways to make LXs. For example, 5-lipoxygenase in neutrophils (i.e., ALOX5) and 15-lipoxygenase-1 in immature red blood cells and reticulocytes (i.e., ALOX15) work in chains to form LxA44 and LxB44. This pathway also occurs in chain interactions between neutrophils and eosinophils, epithelial or M2 macrophages/monocytes and neutrophils, endothelial or skeletal muscle and neutrophils [69,70,71]. As LXs were discovered earlier than other SPMs, derivatives for LXs with increased stability were also synthesized, and studies are currently underway in various inflammation-related indications, including cardiac fibrosis.

#### 4.1.2. Resolvins (Rvs)

Rvs is a specialized pro-resolving mediator that is derived from omega-3 fatty acids, primarily EPA and DHA, docosapentaenoic acid (DPA) and clupanodonic acid. Rvs are divided into several subclasses based on the unique aspects of the structure and the straight-chain polyunsaturated fatty acids (PUFAs) from which they are formed. RvDs are metabolites of 22-carbon PUFA, DHA (i.e., 4Z, 7Z, 10Z, 13Z, 16Z, 19Z)-docosahexaenoic acid.

RvE is a 20-carbon metabolite of EPA (i.e., 5Z, 8Z, 11Z, 14Z, 17Z-5,8,11,14,17-eicosapentaenoic acid); Resolvin Dn-6DPA (RvDsn-6DPA) is a DPA isomer, a metabolite of osbond acids (i.e., 4Z, 7Z, 10Z, 13Z, 16Z-docosapentaenoic acid); Resolvin Dn-3DPA (RvDn-3DPA) is a metabolite of the DPA isomer, clupanodonic acid (i.e., 7Z, 10Z, 13Z, 16Z, 19Z)-docosapentaenoic acid; RvT is a metabolite of clupanodonic acid with 17R-hydroxyl residues, as opposed to RvDsn-3DPA (all have 17S-hydroxyl residues). Because AT-RvDs are synthesized by drug-modified cyclooxygenase 2 enzymes to form 17 (R)-hydroxyl rather than the 17 (S)-hydroxyl residue of RvE, specific isomers of RvD are converted to aspirin-triggered RvDs (AT-RvDs). All mentioned Rvs, except RvDsn-6DPA, are metabolites of omega-3 fatty acids [72,73].

#### 4.1.3. Maresins (MaRs) and Neuroprotectins (NPDs)

MaR1 (7R, 14S-dihydroxy-4Z, 8E, 10E, 12Z, 16Z, 19Z-docosahexaenoic acid) is a member of the SPMs produced in macrophages. MaR1 and recently defined MaR2 are the 12-lipoxygenase-derived metabolites of the omega-3 fatty acid DHA metabolites and they have anti-inflammatory, protective and healing-promoting properties, similar to the other SPM members mentioned earlier in the class. MaR1 was first defined as a product derived from DHA formed by human monocyte-derived macrophage [4]. At the same time, macrophages also convert DHA to 13 (R), 14 (S)-dihydroxy-4Z, 7Z, 9E, 11E, 16Z, 19Z-docosapentaenoic acid—i.e., MaR2 [74].

Neuroprotectin D1 (NPD1) (10R, 17S-dihydroxy-4Z, 7Z, 11E, 13E, 15Z, 19Z-docosahexaenoic acid), also known as Protectin D1 (PD1), is a docosanoid derived from the PUFA docosahexaenoic acid. Like other members of the specialized pro-resolving mediators, NPD1 exerts potent anti-inflammatory and anti-apoptotic/neuroprotective biological activities [75,76]. Other neuroprotective agents with similar activity exist: PDX (10R, 17S-dihydroxy-4Z, 7Z, 11E, 13Z, 15E, 19Z-docosahexaenoic acid); 20-hydroxy-PD1 (10R, 17S, 20-trihydroxy-4Z, 7Z, 11E, 13E, 15Z, 19Z-docosahexaenoic acid); 10-epi-PD1 (10R, 17S-Dihydroxy-4Z, 7Z, 11E, 13E, 15Z, 19Z-docosahexaenoic acid) [77,78]. The activity of the neuroprotectin-like metabolite 17-epi-PD1 (10R, 17R-dihydroxy-4Z, 7Z, 11E, 13E, 15Z, 19Z-docosahexaenoic acid) has not yet been reported.

### 4.2. SPM Receptors

With the discovery of SPMs that promote the termination of inflammation, new receptors that mediate the action of SPMs have also become known, providing opportunities for new drug targets to stop inflammation. The SPMs and their respective SPM receptors, which mediate their actions, are briefly described in this section (Figure 2). Enthusiastic researchers have demonstrated that the pro-resolving activity of SPM occurs through the activation of one or more G protein-coupled receptors (GPCRs) present in the plasma membrane. However, there are still unidentified receptors that mediate the action of some types of SPMs.

Four GPCRs have been reported as receptors for RvD1 and RvE1. However, it has not been confirmed whether other Rvs and PDs, such as RvE2, RvE4, RvD2, RvD3, and PDX, act on these GPCRs [79,80,81]. For the recent and specific physiological actions of these receptors and detailed study data of receptor null mice, please refer to other reviews and references therein [82,83]. There are also receptor molecules that do not exist on the cell membrane but mediate the action of SPMs, which are briefly discussed in Section 4.2.7.

#### 4.2.1. Chemerin Receptor 1

Chemerin1 was initially classified as an orphan GPCR with homology to the formyl peptide receptor and the anaphylatoxin C3a and C5a receptors [52,84,85]. It was not until the early 2000s that it was discovered that the ligand for this receptor was chemerin [86]. In addition to chemerin, RvE1 was identified as a second endogenous agonist through a screening program on the GPCR panel [80]. ChemR23 (a chemokine-like receptor 1, also known as CMKLR1) is a receptor for RvE1, which has been shown to bind more strongly than chemerin (peptide ligand) [79,87]. Besides, RvE2 is a partial agonist compared to RvE1 in CHO-chemerin1 β-arrestin recruitment [88].

Chemerin receptor 1 (chemerin1, ChemR23, or ERV1) is expressed on a wide range of immune cells, including monocytes, macrophages, NK cells, bone marrow cells, and DC. Additionally, ERV1 has been identified in adipocytes and endothelial cells [89]. Expression of chemerin1 is more abundant in antigen-presenting cells (APCs), such as macrophages and dendritic cells than in neutrophils and T-lymphocytes. However, the expression is upregulated in neutrophils in various diseases, including type 2 diabetes [90]. The expression of chemerin is associated with inflammatory and fibrotic processes associated with kidney damage [91]. Serum levels of chemerin were significantly increased in non-alcoholic fatty liver disease patients compared to the controls [92]. However, there appears to be no report of a link to heart fibrosis.

#### 4.2.2. *N*-Formyl Peptide Receptor 2/LXA4 Receptor (FPR2/ALX)

Originally, FPR2 was classified as an FPR receptor due to the activation by the low-affinity endogenous agonist *N*-formyl methionyl peptide (fMLP) [93]. Through the screening of various receptor ligands using radiolabeled [3H]-LXA4 and subsequent GTPase activity, it was confirmed that cells overexpressing FPR2/ALX cDNA (pINF154) exhibit high affinity and specificity for LXA4 binding [94]. Thus, the receptor was reclassified as FPR2/ALX, because LXA4 exhibited the highest affinity of all FPR2/ALX endogenous agonists [95]. The binding of LXA4 induces the stimulation of monocyte chemotaxis, macrophage differentiation and efferocytosis [96,97]. LXA4 upregulates FPR2/ALX by activating the receptor promoter, acting as positive feedback on FPR2/ALX expression [98]. The expression of FPR2/ALX is also markedly increased in the presence of inflammatory stimulants, including tumor necrosis factor-α (TNF-α), platelet-activating factor (PAF), and IL-8 [99].

FPR2/ALX is expressed on leukocytes, including neutrophils, monocytes, macrophages, dendritic cells, naive CD4 T cells, and Th1, -2, -17 cells. FPR2/ALX is also expressed in non-immune cells, including endothelial, keratinocytes and mesenchymal stem cells [100]. Endogenous and exogenous lipids, peptides and proteins can bind and activate FPR2/ALX to produce inflammatory and anti-inflammatory effects [101,102]. The LXs and Rvs families, including LXA4, AT-LXA4 (15-epi-LXA4), RvD1, AT-RvD1 (17-epi-RvD1) and Annexin A1 (ANXA1), all activate receptors with high potency. Meanwhile, serum amyloid A (SAA) and cathelicidin (LL-37) have been identified as endogenous antagonists [103,104].

The positive control of FPR2 can also improve pulmonary fibrosis, dermal fibrosis, and cystic fibrosis [105,106,107]. Impaired inflammatory control after myocardial infarction (MI) promotes left ventricular (LV) remodeling and loss of function. In left ventricle (LV) and splenic remodeling post-myocardial infarction (MI), the RvD1-group showed an early exit of neutrophils from LV and spleen at day 5 after MI with the increased expression of the LXA4 receptor (ALX; synonym formyl peptide receptor; FPR2) compared to the MI-saline group [108]. Cmpd43, a dual FPR1/FPR2 agonist, stimulated FPR1/2-mediated signaling, enhanced pro-resolving cellular function, and regulated cytokines. Cmpd43 also increased the pro-resolving macrophage marker while increasing LV function and reducing chamber remodeling. From these results, it is likely that the positive control of FPR2 by RvD and LXA4, which are ligands of FPR2, can be a significant inhibitory means of general fibrosis, including cardiac fibrosis.

#### 4.2.3. GPR18

GPR18 was discovered as a receptor for RvD2 through GPCR-β-arrestin-based screening and the receptor was called DRV2/GPR18 [109,110,111]. In any case, several other ligands activate DRV2/GPR18. These include endogenous ligands, such as *n*-arachidonylglycine (NAGly), anandamide, a metabolite of the endocannabinoid anandamide, synthetic ligands such as abnormal-cannabidiol (Abn-CBD), and O-1918, a partial agonist. These substances serve as useful pharmacological tools to inhibit DRV2/GPR18 signaling [112,113]. GPR18 is abundantly expressed in polymorphonuclear neutrophils (PMNs), monocytes and macrophages [114]. RvD2 treatment increased the expression of CD163 and CD206, a classic marker of anti-inflammatory macrophages, and the upregulation of GPR18 expression [109,110]. Moreover, the activation of GPR18 by RvD2 reduced lipopolysaccharide (LPS) and ATP-stimulated inflammation in macrophages, and this effect was lost by the GPR18/GPR55 antagonist O-1918 [115]. In patients with sepsis, GPR18 is significantly reduced in PMN, indicating a role in resolving inflammation [116]. To the best of our knowledge, the potential role of GPR18 in the development of fibrosis has not been characterized in any previous study. This gene is known to be involved in the termination of inflammation and, therefore, it is likely to play an essential role in the control of fibrosis.

#### 4.2.4. GPR32

GPR32 is primarily expressed in human PMN, monocytes, adipose tissue and vascular endothelial cells [117]. RvD1 has been identified as a potential agonist due to the activation of GPR32, where [3H]-RvD1 binds to human leukocytes and significantly lowers TNF-α-stimulated NF-κB signaling in GPR32 overexpressing cells [81]. RvD1 has a higher affinity for GPR32 than FPR2/ALX, but its interaction with GPR32 has not been extensively studied [118]. This may be because GPR32 exists as a pseudogene in rodents, making animal testing, in principle, inappropriate.

The treatment of inflammatory macrophages expressing GPR32 with RvD1 enhances the pro-resolving phenotype to increase phagocytosis and reduce the secretion of inflammatory cytokines [119]. Additionally, GPR32 mediates the inhibition of epithelial mesenchymal transition (EMT) in lung cancer cell lines by RvD1 [120]. Anti-GPR32 antibodies reversed the reduction in PMN migration. However, anti-FPR2/ALX antibodies did not show similar effects. After increasing the RvD1 concentration, the effect was reversed, confirming that RvD1 had a higher efficacy against GPR32 than FPR2/ALX [118]. Besides, RvD3, AT-RvD3 and RvD5 have all been shown to activate GPR32 in a recombinant system of β-arrestin recruitment [121,122]. This fact suggests the potential redundancy of ligands acting on GPCRs. GPR32 is involved in the inhibition of TGF-β1-induced EMT by RvD1 in lung cancer cells and primary alveolar type II (ATII) cells [120,123]. In particular, it reduced fibroproliferation and collagen production in ATII cells [123]. These results show that GPR32 may be a target for fibrosis regulation.

#### 4.2.5. GPR37

GPR37 or Parkin-associated endothelin-like receptor (Pael-R) was originally discovered through genomic library screening to find new neuropeptide receptors [124]. The GPR37 receptor is primarily expressed in the brain and is associated with neurological disorders, such as Parkin’s disease, and autism [125]. Mutations within GPR37 affect a variety of autism spectrum disorders, dopamine reuptake regulation, and oligodendrocyte differentiation [126,127,128]. PD1 is considered a ligand for GPR37 because it induced a significant increase in intracellular calcium in HEK293 cells overexpressing GPR37 and in murine peritoneal macrophages [129]. Based on the fact that Gpr37 (-/-) mice exhibited increased apoptosis and infarct size, it has recently been suggested that GPR37 is also involved in cell damage protection and inflammation after ischemic stroke [130]. There seems to be no report of whether GPR37 regulates fibrosis.

#### 4.2.6. Leukotriene B4 Receptor 1 (BLT1)

BLT1 is a receptor for RvE1 and leukotriene B4 (LTB4). However, high-affinity LTB4 is a potent lipid inflammatory chemoattractant, but induces T helper cell chemotaxis and early effector T cell recruitment through BLT1 [80,131]. BLT1 shares 21% sequence identity with chemerin1. Although this value is relatively low, the selective BLT1 antagonist U-75302 has been demonstrated to replace the binding of [3H]-RvE1 to human PMN membranes [80]. The activation of BLT1 by RvE1 also acts as a feedback mechanism for other SPMs, including increased production of LXA4 in the FPR2/ALX-mediated resolution of allergic pulmonary inflammation [132]. RvE2 was identified as an additional BLT1 agonist, and β-arrestin analysis showed that RvE2 blocks LTB4-mediated β-arrestin signaling with similar efficacy to RvE1, indicating direct competition with LTB4 [133]. Various pro-solving roles of RvE2 have been proposed, including the regulation of PMN infiltration and IL-10 production [133]. However, while RvE1 promotes NADPH oxidase-mediated ROS production through the BLT1 receptor, RvE2 and RvE3 do not exhibit this effect [134]. The activation of BLT1 via LTB4 is involved in promoting fibrosis [135,136,137]. Research on whether fibrosis is inhibited by RvE1, a ligand related to the termination of inflammation of BLT1, has not yet been conducted. Exploring this topic would provide insights into the role of BLT1, RvE1, and LTB4 in fibrosis.

#### 4.2.7. Miscellaneous SPMs Receptors

Several studies have reported other GPCR-related possibilities that SPMs activate. Among them, GPR101 mediates the resolving effects of RvD5n-3 DPA in arthritis and infection [138]. Furthermore, SPMs has been reported to activate non-GPCR receptors such as nuclear receptors. Dose-dependently, PD1 enhances peroxisome proliferator-activated receptor γ (PPAR-γ) transcription-activated reporter activity in human neuron-glia (HNG) cells co-transfected with hPPAR-γ-GAL4 and MH100-tk-luc [139]. The transcriptional activity of PPAR-γ increased significantly after treatment with 100 nM PD1. This suggests that PD1 may enhance the PPAR-γ. RvD1 was also hypothesized as a ligand for PPAR-γ. It inhibited IκBα degradation and NF-κB p65 nuclear translocation in an LPS-induced lung injury model, which was partially reversed by the PPAR-γ inhibitor GW9662 [140]. Interestingly, PPAR-γ has the action of improving fibrosis, including cardiac fibrosis [141,142]. GPCRs that act directly on MaR1 have not yet been identified. However, MaR1 blocks TRPV1-mediated currents in neurons, acts as a ligand for the retinoid-associated orphan receptor α (RORα), and inhibits TLR4 signaling [143], Chiang et al. found that MaR1 can activate leucine rich repeat containing G protein-coupled receptor 6 (LGR6), a member of the glycoprotein hormone receptor subfamily of this rhodopsin-like GPCR. LGR6 initiates cAMP, impedance changes and stimulates innate immune responses to PMN, monocytes and macrophages [144].

## 5. Specialized Pro-Resolving Lipid Mediators and Cardiac Fibrosis

There were not many reports on the improvement of cardiac fibrosis by SPMs resulting from DHA and EPA. As mentioned above, in many papers, the activation of NRF2 has been reported as a major mechanism of action for improving cardiac fibrosis. There have been many reports on the relationship between NRF2 activation and SPMs. Thus, in this section, we will first describe the relationships between NRF2 and DHA, EPA, and SPMs.

### 5.1. Connection between NRF2 and SPMs

We mentioned earlier that activation of NRF2 in cardiac fibrosis can improve cardiac fibrosis (see Section 3.2). This section summarizes the relationship between the DHA, EPA, and SPMs reported so far and NRF2.

#### 5.1.1. Connection between NRF2 and DHA/EPA

What is the relationship between DHA and EPA and SPMs derived from them with NRF2? Antarctic krill oil (AKO) contains abundant EPA and DHA, accounting for more than 27% of its total fatty acid profile [145]. The serum levels of EPA and DHA, and relative protein levels of KEAP1, and NRF2 in the peripheral blood leukocytes from intervention group (IG) patients taking AKO were higher than those in the control group (CG) of patients not taking the AKO (*p* < 0.05) [145]. Serum levels of reactive oxygen species (ROS), 8-hydroxy-2-deoxyguanosine (8-OHdG), nitric oxide (NO) and malondialdehyde (MDA) of the IG group were lower than those of the CG group. The levels of superoxide dismutase (SOD), glutathione (GSH) and glutathione peroxidase (GPx) in the IG group were higher than in the CG group (*p* < 0.05) [145].

Omega-3 polyunsaturated fatty acids exert antioxidant effects through the NRF2 pathway in immortalized mouse Schwann cells [146]. The mechanism of action appears to be that the n-3 fatty acid oxidation product destabilizes the bond between Keap1 and Cullin3, thereby activating NRF2 [147]. Omega-3 polyunsaturated fatty acids also showed antioxidant effects in 3T3-L1 adipocytes and murine astrocytes through the NRF2/HO-1 pathway [148,149]. Omega-3 fatty acids activate NRF2 and upregulate HO-1 to protect the brain from ischemic damage [150]. N-3 PUFA induces inflammatory resistance through the formation of KEAP1-containing cytosolic speckles of the autophagic receptor SQSTM1/p62 (sequestosome 1) SQSTM1/p62-body and activation of NFE2L2 [151]. n-3 PUFA induces acute myeloid leukaemia cell death associated with mitochondrial glycolytic switch from oxidative respiration and the activation of the NRF2 pathway [152]. EPA treatment also increased NRF2 nuclear translocation and antioxidant activity, leading to the upregulation of HO-1 expression, and treatment with EPA reduced apoptosis due to hydrogen peroxide [153]. EPA regulates NRF2 to prevent oxidative stress and inflammatory responses caused by tetrachlorodibenzo-p-dioxin (TCDD) [154]. EPA prevents salt sensitivity and reduces oxidative stress in diabetic mice [155].

17-Oxo-docosahexaenoic acid induces the NRF2-mediated expression of HO-1 in mouse skin and cultured rat epidermal cells in vivo [156]. DHA increases the expression of oxidative stress-induced growth inhibitor 1 through the PI3K/AKT/NRF2 signaling pathway in breast cancer cells [157]. Marine n-3 PUFA DHA induces autophagy and NFE2L2 in human retinal pigment epithelial cells, leading to cellular protection against oxidative stress and protein misfolding [158]. The inhibition of inflammation by DHA is, in part, through communication between the NRF2/HO-1 and the IKK/NF-κB pathway [159]. DHA protects against traumatic brain injury by regulating NADPH oxidase 2 production through the NRF2-ARE signaling pathway [160,161]. DHA and MeHg treatment markedly induced the expression of endoplasmic reticulum (ER) stress markers’ (CHOP and DNAJB9) and NRF2 target genes’ (p62 and HMOX-1) mRNA levels and, unexpectedly, adding EPA to DHA and MeHg treatment attenuated DHA and MeHg-induced apoptosis and inhibited ER stress and the expression of NRF2 target genes [162]. DHA can also block nerve damage and prevent stroke, and its inhibitory effect on nerve cell damage is achieved through DHA’s antioxidant (through induction of the NRF2/HO-1 system) and anti-inflammatory effects (through JNK/AP-1 signaling) [163].

#### 5.1.2. Connection between NRF2 and LXs

**Figure 4:**
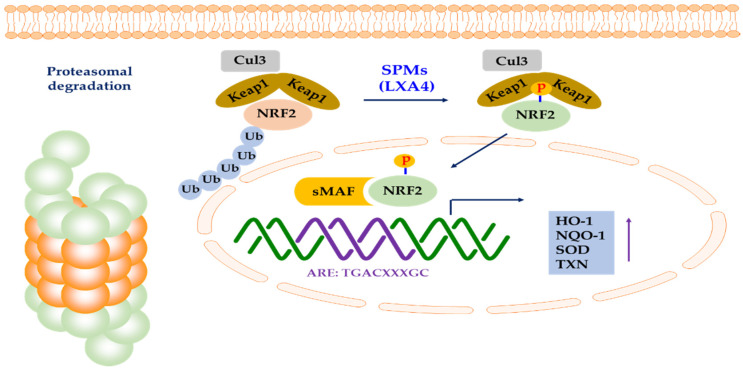
Activation of NRF2 by SPMs. LXA4 can activate NRF2 by inducing the phosphorylation of Ser40 of NRF2 to induce nuclear translocation. Phosphorylated NRF2 can form a heterodimer with sMAF and bind to ARE, leading to the transcription of antioxidant genes, such as HO-1, NQO-1, SOD, and TXN (modified from Lin et al. [173] and Hiebert et al. [52]).

LXA4-induced HO-1 protects cardiomyocytes from hypoxia/reoxygenation damage through p38 MAPK activation and NRF2/ARE complexes [164]. NRF2 is translocated to the nucleus and binds to the HO-1 ARE and E1 enhancer, leading to upregulation of HO-1. LXA4 inhibited oxidative stress-induced vascular endothelial cell (EC) damage and the expression of thrombosis-related factors by the same mechanism [165]. LXA4 inhibits EC hyperpermeability induced by LPS in human umbilical vein endothelial cells (HUVEC), and NRF2 is also involved [166]. LXA4 also exhibits various pharmacological actions through NRF2 in nerve-related cells. In other words, it improves cellular damage through NRF2 in cultured cortical astrocytes exposed to glucose deficiency/reperfusion stimulation [167]. Besides, LXA4 upregulates NRF2 to protect against brain ischemia-reperfusion injury and spinal cord injury [168,169,170]. LXA4 induced NRF2 expression and nuclear translocation, HO-1 expression, and GSH synthesis. LXA4 reduced LPS-induced mouse acute lung injury through NRF2-mediated E-cadherin expression [171]. LXA4 further promoted the dissociation of NRF2 and Keap1 in LPS-treated 16HBE cells, and LXA4 activated NRF2 by inducing the phosphorylation of Ser40 of NRF2 to induce nuclear translocation (Figure 4). LXA4 also improved acute lung injury related to acute pancreatitis through the antioxidant and anti-inflammatory effects of the NRF2 pathway [172]. In Nrf2-/- mice, the effect of LXA4 on the reduction in inflammatory factors was partially attenuated [172]. This result suggested that NRF2 is involved in the LXA4-induced reduction in the level of inflammatory factors.

LXA4 preconditioning attenuates intestinal ischemia-reperfusion injury via the KEAP1/NRF2 pathway in a LXA4 receptor-independent manner [174]. The LXA4 receptor antagonist Boc-2 reversed the protective effect of LXA4 on intestinal damage but did not affect oxidative stress and the associated NRF2 pathway [174]. Additionally, the NRF2 antagonist brusatol reversed the antioxidant effects imparted by LXA4, exacerbating oxidative stress and apoptosis in intestinal epithelial cells, eventually reducing the survival rate of mice [174]. LXA4 treatment upregulated p38 MAPK activation, Nrf2 nuclear translocation, and the binding activity of NRF2 to HO-1 ARE and E1 enhancers in human kidney cells exposed to H/R injury [175]. The treatment of bone marrow-derived macrophages with 15-epi-LXA4 (e-LXA4) inhibits the LPS-dependent activation of NF-κB and IκB kinase β activities, protects against LPS activation-dependent apoptosis, and enhances the accumulation of NRF2 transcription factors. Moreover, the treatment of LPS-stimulated bone marrow-derived macrophages with e-LXA4 dramatically reduces the Kv current, which is compatible with the attenuation of the inflammatory response [176].

LXs were discovered before other SPMs, resulting in synthetic derivatives with increased chemical stability, and the possibility of NRF2 involvement in pharmacological action by these compounds is suggested. In other words, LXA4 methyl ester activates the ERK/NRF2 signaling pathway in rats, improving cognitive impairment due to chronic decreased cerebral perfusion [167]. The LXA4 Receptor/FPR2 Agonist BML-111 protected mouse skin from ultraviolet B radiation, and an increase in NRF2 signaling was observed [177]. BML-111 treatment prevents cardiac cell death and oxidative stress in an autoimmune myocarditis mouse model [178]. These beneficial effects were mediated by the activation of the NRF2 pathway via the CaMKK2-AMPKα kinase pathway. BML-111 also attenuates high glucose-induced inflammation, oxidative stress, and reduces extracellular matrix accumulation by targeting NRF2 in murine glomerular media cells [179]. Finally, BML-111 exerts anti-inflammatory effects in a chronic obstructive pulmonary disease (COPD) mouse model by preventing NLRP3 inflammasome activation and inhibiting ROS production through NRF2 upregulation [180].

#### 5.1.3. Connection between NRF2 and Rvs

RvD1 prevents memory impairment and hippocampal damage in mice fed a corn oil-based high fat diet through NRF2 upregulation and p66 Shc downregulation and inactivation [181]. RvD1 attenuates ventilator-induced lung damage by reducing HMGB1 release in the HO-1 dependent pathway [182]. At this condition, RvD1 increases the expression of NRF2 and inhibits the activation of NF-κB. AT-RvD1 inhibited TGF-β1-induced EndMT (endothelial to mesenchymal transition) by increasing the expression of Smad7 [183]. AT-RvD1 restored the decrease in NRF2 by TGF-β1 and decreased the increase in vimentin expression. AT-RvD1 recovers mouse lungs after tobacco smoke-induced emphysema through downregulation of oxidative stress by the NRF2/KEAP1 pathway [184]. AT-RvD1 also alleviated paraquat-induced acute lung injury in mice [185]. At this time, AT-RvD1 activated NRF2 and upregulated the expression of its downstream genes (NQO-1 and HO-1). This action was also observed when Rvs suppressed skin inflammation and UV-induced oxidative stress [186]. Besides, RvD1 promotes corneal epithelial wound healing and mechanical sensation recovery in diabetic mice [187].

#### 5.1.4. Connection between NRF2 and MaRs

MaR1, another member of SPMs, promotes the nuclear translocation of NRF2 in Sprague–Dawley mice, improves hepatic ischemia-reperfusion injury and stimulates hepatocyte proliferation [188]. MaR1 also modulates the NRF2 and TLR4/NF-κB signaling pathways to relieve dextran sulfate sodium-induced ulcerative colitis [189]. MaR1 activated NRF2 signaling and reduced TLR4/NF-κB activation, and ML385, an inhibitor of NRF2, significantly suppressed these effects of MaR1. MaR1 alleviates renal ischemia/reperfusion injury in mice through inhibition of the TLR4/MAPK/NF-κB pathway and activation of the NRF2 pathway [190]. MaR1 decreased the nuclear translocation of NF-κB and increased the nuclear translocation of NRF2 in this model. MaR1 also improves pulmonary ischemia/reperfusion injury by inhibiting oxidative stress through the activation of the NRF2 mediated HO-1 signaling pathway [191]. Specifically, MaR1 significantly reduces the production of ROS, methane dicarboxylic acid aldehyde and 15-F2t-isoprostane, and restores antioxidant enzymes (superoxide dismutase, glutathione peroxidase and catalase) activity. This appears to be through the expression of nuclear NRF2 and cytoplasmic HO-1.

### 5.2. Therapeutic Effects of DHA and EPA, and SPMs on Cardiac Fibrosis

#### 5.2.1. Therapeutic Effects of DHPA and EPA on Cardiac Fibrosis

Before examining the effects of SPMs on cardiac fibrosis, we would like first to mention the effects of DHA and EPA on cardiac fibrosis. DHA and EPA do not only impact cardiac fibrosis but show improvement in various fibrosis. In other words, for example, krill oil (KO), which is high in DHA and EPA, relieves oxidative stress, iron accumulation and fibrosis in the liver and spleen of iron-overloaded mice [192]. In clinical trials, high-dose EPA (4 g/day) reduced the incidences of clinical events by 25% in statin-treated patients with established CVD or diabetes and other cardiovascular risk factors [193]. The cardioprotective effect of omega-3 fatty acids and ascorbic acid improves the regenerative capacity of embryonic stem cell-derived cardiac lineage cells [194]. Pre-incubation of cells with EPA + DHA + AA before H_2_O_2_ treatment weakened the production of reactive oxygen species (ROS), improved cell viability and increased HO-1 and CX43 (connexin43) expression, leading to a marked rise in cardiac troponin (TNNT2) positive cells and a decrease in vimentin-positive cells [194]. In particular, the injection of EPA + DHA + AA pretreated ESC (embryonic stem cell)-derived cardiac lineage cells into the ischemic myocardium of the MI rat model significantly reduced fibrosis compared to the vehicle group [194].

FO, which is rich in omega-3 polyunsaturated fatty acids, reduced the arachidonic acid (AA) to DHA plasma ratios [195]. In the rat diabetes model (DM), FO reduced cardiac nitrite and MPO and plasma ET-1 levels and increased cardiac glutathione, catalase, and SOD activities. FO prevented DM-related cardiac fibrosis and reduced TGF-β1 and p38 MAP kinases in the heart [195]. ω3-PUFA exhibits cardioprotective action by signaling through the free fatty acid receptor 4 (Ffar4), a G-protein coupled receptor (GPR) for long-chain fatty acids (FA) [196]. EPA, not DHA, prevents fibrosis in heart failure due to pressure overload: a potential role of Ffar4 is suggested [197]. The superphysiological levels of ω3-PUFA achieved by 12 weeks of dietary supplementation prevented fibrosis and diastolic function following pressure overload (transverse artery contraction (TAC)), which was meant to mimic heart failure with preserved ejection fraction [198].

DHA reverses AngII-induced RECK inhibition and migration of cardiac fibroblast [198]. EPA and DHA reversed AngII-mediated RECK inhibition, and DHA appeared to be more effective. EPA and DHA reversed AngII-induced miR-21 expression, RECK inhibition, MMP2 induction, and cardiac fibroblast migration. Therefore, it may exert its potential beneficial effects in cardiac fibrosis. Treatment with omega-3 fatty acids inhibited the loss of CX43 protein by IL-1β, and more importantly, suppressed the loss of CX43 function by inhibiting the translocation of NF-κB. Diets rich in omega-3 fatty acids in the intact heart limited the loss of CX43 in the disc inserted into the heart after MI [199].

DHA supplementation reduced susceptibility to atrial fibrillation, and in dogs, DHA also reduced atrial fibrosis compared to No-PUFA [200]. Long-term administration of EPA improves cardiac remodeling after myocardial infarction in mice by modulating macrophage polarization [201]. Highly purified EPA improves heart damage and adipose tissue inflammation in a rat model of metabolic syndrome [202]. EPA suppresses the side effects of C-reactive protein overexpression on cardiac remodeling due to pressure overload [203]. EPA prevents atrial fibrillation associated with heart failure in rabbit models [204].

#### 5.2.2. Therapeutic Effects of SPMs on Cardiac Fibrosis

Compared to the effects of DHA and EPA on improving cardiac fibrosis, there are few reports on the therapeutic effect on cardiac fibrosis by pro-resolving lipids mediators derived from them. Chagas disease, caused by the protozoan *Trypanosoma cruzi*, progresses to chronic progressive inflammatory cardiomyopathy, which leads to heart failure and death during the acute asymptomatic stage if an appropriate treatment is not administered [205]. Statins can regulate chagasic myocarditis by inducing the production of 15-epi-lipoxin A4 (15-epi-LXA4), a pro-resolving lipid mediator in inflammatory cardiomyopathy. Ticagrelor, which acts as a platelet aggregation inhibitor by antagonizing the P2Y12 receptor [206], protects the heart from reperfusion injury and improves remodeling after myocardial infarction [207]. Ticagrelor alleviated fibrosis and decreased collagen-III mRNA levels in ischemia/reperfusion models [207]. Besides, ticagrelor attenuated the increase in pro-inflammatory tumor necrosis factor-α, interleukin-1β, and interleukin-18, and increased anti-inflammatory 15-epi-LXA4 levels, which suggested that 15-epi-LXA4 was involved in ticagrelor-induced improving cardiac fibrosis [207].

BML-111 treatment prevents cardiac cell death and oxidative stress in an autoimmune myocarditis mouse model [178]. In vivo and in vitro studies have demonstrated that these beneficial effects are mediated by the activation of the NRF2 pathway via the CaMKK2-AMPKα kinase pathway. Cardiac dysfunction, hypertrophy, and neofibrosis found in experimental autoimmune myocarditis mice were prevented by BML-111 treatment [178].

RvD1 improves ventricular function by activating the pro-resolving response in the ventricular region after myocardial infarction [108]. In this method, 8–12-week-old male C57BL/6J mice were coronary ligated and injected with Lipo-RvD1 or RvD1 (3 μg/kg/day) for 3 h after MI (d) for 1 day or until fifth day. The RvD1-group showed increased expression of the LXA4 receptor (ALX; synonym formyl peptide receptor; FPR2) compared to the MI-saline group. RvD1 stabilized the extracellular matrix by reducing the pro-fibrotic genes (colla1, coll2a1 and tnc (all; *p* < 0.05)) and reducing collagen deposition, thereby reducing fibrosis after MI.

## 6. Conclusions

Cardiac fibrosis is a serious disease that is difficult to cure. DHA and EPA have therapeutic effects on cardiac fibrosis through the activation of NRF2. Recently, the collection of results on SPMs produced from DHA and EPA have proved the potential against various chronic inflammatory diseases. Cardiac fibrosis, in which inflammation plays an important role, can also be such a disease. Among the SPMs, BML-111, an LX derivative, and RvD1 have been effective against cardiac fibrosis, but many other SPMs, such as RvEs, MaRs, and PDs, have not been reported with regard to their efficacy in treating cardiac fibrosis. This is likely not because SPMs may not have a therapeutic effect on cardiac fibrosis, and because it is not easy to get enough SPMs to evaluate the therapeutic effects for cardiac fibrosis.

There are many reports that DHA and EPA, the precursors of SPMs, effectively treat cardiac fibrosis through the activation of NRF2 and that SPMs can activate the NRF2. Therefore, it is expected for other SPMs to show a therapeutic effect. Accordingly, there are many reports that SPMs are already effective in fibrosis in other organs, such as lung. Therefore, the therapeutic effect of SPMs via NRF2 in cardiac fibrosis is predicted to be sufficiently effective, and studies on this prediction are expected to proceed actively. In particular, the activation of NRF2 through novel SPMs and their derivatives seems to be a promising new strategy for the treatment of cardiac fibrosis.
